# Designing a multifaceted quality improvement intervention in primary care in a country where general practice is seeking recognition: the case of Cyprus

**DOI:** 10.1186/1472-6963-8-181

**Published:** 2008-08-27

**Authors:** George A Samoutis, Elpidoforos S Soteriades, Henri E Stoffers, Theodora Zachariadou, Anastasios Philalithis, Christos Lionis

**Affiliations:** 1 Clinic of Social and Family Medicine, School of Medicine, University of Crete, Heraklion, Crete, Greece; 2 Department of Primary Care, Cyprus Institute of Biomedical Sciences (CIBS), Nicosia, Cyprus; 3 Department of Environmental Health, Environmental and Occupational Medicine and Epidemiology (EOME), Boston, Harvard School of Public Health, MA, USA; 4 Department of General Practice, School of Primary Care and Public Health (CAPHRI), Maastricht University, Maastricht, The Netherlands; 5 Health Planning Unit, School of Medicine, University of Crete, Heraklion, Crete, Greece

## Abstract

**Background:**

Quality Improvement Interventions require significant financial investments, and therefore demand careful consideration in their design in order to maximize potential benefits. In this correspondence we present the methodological approach of a multifaceted quality improvement intervention aiming to improve quality of care in primary care, properly tailored for a country such as Cyprus where general practice is currently seeking recognition.

**Methods:**

Our methodological approach was focused on the design of an open label, community-based intervention controlled trial using all patients from two urban and two rural public primary care centers diagnosed with hypertension and type II diabetes mellitus. The design of our intervention was grounded on a strong theoretical framework that included the Unified Theory of Acceptance and Use of Technology, and the Chronic Care Model, which synthesize evidence-based system changes in accordance with the Theory of Planned Behavior and the Theory of Reasoned Action. The primary outcome measure was improvement in the quality of care for two chronic diseases evaluated through specific clinical indicators, as well as the patient satisfaction assessed by the EUROPEP questionnaire and additional personal interviews.

**Results:**

We designed a multifaceted quality improvement intervention model, supported by a varying degree of scientific evidence, tailored to local needs and specific country characteristics. Overall, the main components of the intervention were the development and adoption of an electronic medical record and the introduction of clinical guidelines for the management of the targeted chronic diseases facilitated by the necessary model of organizational changes.

**Conclusion:**

Health planners and policy makers need to be aware of the potential use of certain theoretical models and applied methodology as well as inexpensive tools that may be suitably tailored to the local needs, in order to effectively design quality improvement interventions in primary care settings.

## Background

The quality of health care services, as measured by standardized indicators and stakeholders' satisfaction, constitutes a cornerstone of health care delivery in the current era of continuous health care reform. However, the achievement of quality of care remains a challenge for many western societies, despite a continuously increasing level of health care expenditure [1]. Furthermore, many efforts in the hospital setting have been devoted to limit practice variation, effectively utilize available electronic resources and improve patient satisfaction; whereas in primary care services the importance of financial savings along with significant health gains has not been adequately explored. In our opinion, high health care quality requires universal access, equity in services and cost-effective care [2]. In particular, improving chronic illness management has been attracting increasing interest by health care providers and government agencies, since the average chronic patient entering the primary care system is not receiving an optimal quality of care [3]. Furthermore, the ageing population, predominantly in the western societies, constitutes an ever growing economic and health care burden requiring increasing community-based services [4].

A number of different quality improvement (QI) interventions implemented at various clinical settings have been evaluated and presented in the medical literature [5-8]. Such interventions range from single-component approaches (e.g. electronic reminder systems) to multifaceted complex strategies combining both patient-mediated activities (e.g. educational leaflets) and health care provider services (e.g. consensus building, training, and audit/feedback processes) [9]. In addition, tailoring interventions to the local group practitioners' needs, has also been proposed as a concerted effort to attain successful and sustainable outcomes compared to interventions that are fixed and lack programmatic flexibility [10]. Moreover, interventions for quality improvement involve significant amounts of financial investment, and therefore require careful modeling in order to maximize potential successes [11]. Finally, process and outcome measure evaluation of such attempts is thought to be of paramount importance in order to assist decision makers in developing appropriate policies for structural and long-term planning.

Many countries with adequate resources, high capacity in primary care research and past experience in quality improvement efforts, have been actively involved in implementing major modifications in primary health care services in order to incorporate quality indicators based on a broad array of methodological strategies [12,13]. The term 'clinical governance' has been introduced to capture a range of activities required to improve the quality of health care services, including the development of processes for continuous monitoring and accountability systems for delivered quality care [14]. However, countries with little experience and limited resources, including Cyprus, face significant challenges in attempting to design QI interventions tailored to country-specific characteristics as described below.

Cyprus is currently moving towards the introduction of a primary care driven, universal health care coverage system for the entire population, with quality improvement processes as an important incorporated component. However, for the time being, Cyprus operates in a dual system of health care delivery, offering publicly-funded health care services to low and medium-income citizens, while the rest, mostly well-off part of the society, utilize services from the private sector, covering their expenses either from private health insurance schemes or through out of pocket compensation [15]. The majority of public as well as private primary care settings are characterized by under-use of contemporary information technologies, limited monitoring systems, and variable use of clinical standards of care such as chronic disease management guidelines and patient satisfaction surveys [16]. The Cyprus Ministry of Health is being strongly interested in investing significant resources to promote quality improvement strategies in both sectors of health care services. Within an already existing framework of collaboration between the Clinic of Social and Family Medicine of the University of Crete and the Cyprus Ministry of Health, a pilot intervention for quality improvement in two public primary health care centers in Cyprus was developed. In this correspondence, a detailed description is presented of the theories and methodology used to design a country-specific quality improvement intervention in an urban and rural primary health care center in Cyprus, tailored to local practices and primary care professionals' needs.

### The Theoretical Framework

Our approach was designed to follow the steps of contemporary theories in order to explore different pathways including the use of information technology and the introduction of standard guidelines for chronic disease management. A literature review and an analysis of the existing organizational and operational context led to the identification of three main areas that required strong theoretical background for a successful design of our intervention; namely: a) an organizational change paradigm; b) the introduction of information technology, and c) the adoption of clinical guidelines into everyday practice for chronic disease management.

#### Organizational Change Paradigm

In anticipation of advanced needs for structural changes in the primary care centers during the intervention, a model of organizational change was adopted, which identifies seven stages: sensing of unsatisfied demands on the system, the search for possible responses, evaluation of alternatives, decision to adopt a course of action, initiation of action within the system, and implementation and institutionalization of a change [17].

#### Information Technology

The introduction of an electronic medical record (EMR) followed the four constructs of the *Unified Theory of Acceptance and Use of Technology *(UTAUT), as described in the current literature: performance expectancy, effort expectancy, social influence and facilitating conditions [18,19]. The UTAUT has been validated empirically amongst four businesses from various industries, and was cross-validated using data from another two businesses, enabling researchers to explain up to 70% of technology acceptance behavior [20]. Based on the above, a UTAUT model was adopted for the design of our intervention due to its comprehensive character and high explanatory power. According to the UTAUT model, technology acceptance depends on: a) user determinants (e.g. age, gender, experience, and voluntariness of use), b) information technology expectancy (e.g. performance expectancy and effort expectancy), c) implementation setting and user professional environment, including social influence, and d) organizational facilitating conditions. All of the above were taken into consideration during the preparedness phase of the project as well as for the planned daily interactions of the improvement team, in order to enable physicians and nurses to successfully adopt the use of EMR in a previously computer-naïve professional environment.

#### Introducing disease management guidelines

The selection of chronic illnesses that were used in the intervention was based on the most common diseases encountered in public primary care centers of Cyprus, namely hypertension (HTN) and type II diabetes (T2DM) [21]. The introduction of clinical guidelines and continuing medical and nursing education for chronic disease management was based on the *Chronic Care Model *(CCM) [22,23], the *Theory of Planned Behavior *(TPB), and the *Theory of Reasoned Action *(TRA) [24,25]. The CCM model synthesizes evidence-based system changes leading to improved outcomes. It emphasizes six main components: a) the organization of health care, b) community linkages, c) self-management support, d) delivery system design, e) decision support and f) information systems.

Furthermore, our design was influenced by the Chronic Care Model in order to implement disease management guidelines, electronic reminder systems, and e-library resources as part of the decision support systems. Moreover, a chronic care support was organized through the implementation of a referral scheme, an electronic appointment scheduling, and the introduction of an electronic medical record system (EMR). In addition, patient education activities were scheduled to be deployed through training with educational materials and face to face guidance for disease self-management (e.g. blood glucose monitoring and foot examination). The Theory of Planned Behavior and Theory of Reasoned Action provided the necessary theoretical framework in order to assist us in empirically identifying those QI intervention factors on which our efforts should be targeted. Application of TPB and TRA models also helped us identify, through the conduction of in-depth open-ended elicitation interviews, the underlying beliefs that determine health professional's attitudes, subjective norms, and perceived behavioral control. Thereby, such theories may potentially affect the health professional's likelihood of modifying previous behaviors and successfully adopting newly introduced clinical guidelines.

### Methodological Hypotheses

Based on the above-described theories and an extensive literature review, we developed specific research hypotheses in accordance with our research model. First, with regards to EMR introduction, we hypothesized that (a) performance expectancy, effort expectancy, and social influence would positively affect Primary Care Physicians' (PCPs) and nurses' attitudes toward adopting EMR technology; (b) organizational facilitating conditions should have a direct effect on performance expectancy, effort expectancy, and health professionals' EMR utilization behavior; and (c) behavioral intention will have a significant positive influence on health professionals' practice.

In addition, with respect to the adoption of guidelines, we expected that (a) PCPs and nurses would accept and effectively implement clinical practice guidelines on chronic disease management; (b) the quality of primary care services for chronic diseases would be improved following the implementation of our intervention; and (c) the use of CCM, TPB and TRA, would help us shape a positive impact on chronic disease management.

Finally, we hypothesized that the proposed design, according to given resources and other de facto local characteristics, such as limited technological adoption, computer-naïve environment, lack of previous experiences with quality improvement interventions, and scarce incentives for performance, would help us efficiently examine the effectiveness of a QI intervention in Cyprus.

## Methods

### A Multifaceted Quality Improvement Intervention in Cyprus

The translation and implementation of the above described theories and models into a busy day-to-day clinical practice, represents a formidable challenge. Common experience with other quality improvement interventions [26] coupled with the above described insights from industrial examples, suggest that sustained improvements in chronic illness care require a comprehensive, continuous, and systematic change approach following a specific intervention. Our operational model was based on a multifaceted intervention that was facilitated through a multidisciplinary quality improvement team.

Current literature supports the implementation of multifaceted interventions in the health care sector, since many components may interact and reinforce each other in encouraging the change of professional practice and promoting workplace satisfaction [27]. Our multifaceted intervention was designed to involve several implementation strategies including a combination of educational components (educational materials, workshops, local opinion leaders' presentations, academic detailing), audit and feedback, and an electronic decision support system enabled through e-library and electronic reminder system tailored to the local needs. Particular tailoring characteristics took into consideration the lack of motivators in the public primary care centers, the scarcity of use of clinical guidelines in daily practice, the absence of referral and appointment systems as well as other organizational weaknesses and the existence of a computer naïve environment. Thus, we incorporated the use of physician facilitators, who introduced several non-monetary incentives for the health professionals, provided them with practical tools such as foot examination screening checklist, and developed tailored organizational changes. We also promoted a strong theoretical framework consisting mainly of the UTAUT and CCM supporting the computer naïve environment, and appointed a responsible individual who had to identify specific resources at each center including the support of a new appointment and referral system. Finally, an informed consent form was developed in order to be used during the implementation phase. The study was approved by the National Bioethics Committee.

#### Organizational Changes

Structural and organizational changes were employed, as equally important components in designing a quality improvement intervention for the management of disease co-morbidities, along with the introduction of standard clinical guidelines [28].

Consensus building meetings were planned in order to identify potential barriers and evaluate alternatives for the introduction of an appointment-based electronic scheduling system, secure continuity of secretarial support during and after the project ended, and introduce an effective specialist referral system. After reaching a consensus, the adopted decision along with necessary organizational changes could take place awaiting the institutionalization of the selected changes. Additionally, a specific plan was applied through a framework of changes that would guide health professionals in their everyday practice. Upper management support from the administrative health services of the Ministry of Health was actively sought.

#### Introduction of Electronic Medical Record (EMR)

The introduction of the EMR system, which was based on the International Classification System for Primary Care (ICPC-2), consisted of the introduction of a windows-based software program (Transhis) described in detail elsewhere [29]. The secretaries, nurses and physicians at the intervention primary care centers were provided with personal computers, printers and a high-speed broadband internet access for all. Primary care physicians (PCPs) and nurses were asked to serve as evaluators of the EMR system performance. In addition, 18 randomly selected patients, half of which were males and half females, were scheduled to undergo personal interviews in order to provide detailed feedback on their experience with the EMR and identify barriers in its daily implementation. Among many other software programs, *Transhis*, a windows-based EMR system, incorporating episode of care and reminder systems was selected to serve as the supporting electronic interface based on defined criteria for appropriateness, efficiency, and feasibility for the general clinical practice [30].

#### Chronic Disease Clinical Guidelines

Chronic disease guidelines represented our decision support tool, one of the main components of the Chronic Care Model. Such a tool was scheduled to provide evidence-based clinical information to the health care professionals that were readily available through electronic databases facilitated by EMR [31]. Type II diabetes mellitus guidelines were based on the American Diabetes Association and St. Vincent Declaration guidelines. Hypertension management guidelines were based on the VII Report of the Joint National Committee, the European guidelines on cardiovascular disease prevention and the European Society of Hypertension – European Society of Cardiology Guidelines, whereas lipid control practice guidelines were based on the National Cholesterol Education Program III.

#### Quality Improvement Team

Setting up a multidisciplinary quality improvement team was thought to be of paramount importance for the successful facilitation and fide implementation of our intervention. The use of a quality improvement team has been described long ago by industrial quality experts [32]. Our study team consisted of two family physicians serving as program facilitators, three academic experts in family medicine with previous extensive experience in QI interventions, and one family physician with expertise in EMR.

Both study facilitators were scheduled to meet with providers in their practice settings during regular time intervals in order to undertake the following actions: (a) record baseline characteristics of participants in the study and present quality indicators for each selected illness; (b) facilitate consensus building for the management of selected illnesses, according to given clinical guidelines; (c) assist in the development and adaptation of tools and strategies for implementing the intervention; (d) facilitate meetings to assess progress and potential barriers in the implementation of the intervention while being able to modify the plan accordingly; (e) conduct interviews of the participating doctors, nurses and patients; and (f) develop and complete electronic chart audits, monthly reports and outreach visit forms.

### Setting and Participants

#### Primary Health Care Centers

The study was scheduled to take place in Nicosia, the capital of Cyprus, in two urban and two rural public primary health care centers (PHCC), which were selected based on population served and employee criteria (age, duration of medical education, number of years in practice). One rural and one urban center were designed to serve as controls being observed to follow regular practice. Each center was designed to have a worksite leader who would leverage resources and be the primary contact person in collaboration with the quality improvement team. All PCPs and nurses from the intervention primary care centers were scheduled to participate in the study as evaluators of the intervention.

#### Patients and Eligibility Criteria

Several reasons supported the patient selection criteria including our objective to include a relatively small, however homogeneous patient population that has also been identified in the medical literature as a frequently neglected group of patients [33]. In addition, HTN and T2DM were found to be the most common diseases in the primary care system of Cyprus [21]. These health problems often occur in a concurrent fashion and lead to serious complications that may not be optimally treated [34,35]. Finally, despite recommendations for more aggressive hypertension therapy in the presence of coexisting diabetes, it is unclear whether there are any differences in how clinicians manage blood pressure in hypertensive patients with or without diabetes [36].

### Study Design

**Figure 1:**
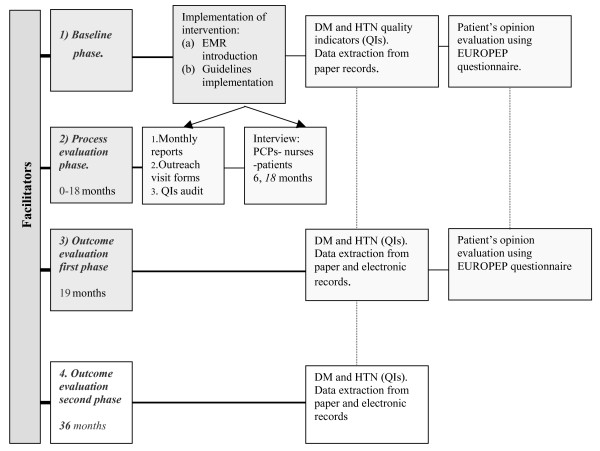
Schematic Representation of the Evaluation Framework.

The proposed model was planned to be evaluated through a community-based open-label intervention control trial comparing regular practice to an EMR-enhanced practice aided by chronic disease management based on standard clinical guidelines. The design included three phases of evaluation including: (a) a baseline assessment, (b) an end of follow-up comparison and (c) an 18-month post intervention evaluation. A window of 3 months run-in-phase was planned for the eligible patients to enter the study. Due to the possibility that deviations from the protocol during the intervention could impact the validity of the trial, we proposed the proper handling and reporting of any non-adherence to the protocol events. In addition, our study design included process and outcome evaluation as depicted in Figure 1[37,38], along with a brief economic analysis (accumulated cost of the personnel, equipment and the intervention itself).

### Process Evaluation

#### Structured forms

The facilitators were expected to complete two structured forms: monthly reports (MR) and outreach visits reports (OVR), which were developed based on previous report from the literature [39]. Monthly reports were expected to provide detailed information on the recording of visits to a Primary Care Health Center (PCHC), the activities within each PCHC, the outcomes of those activities, the number of hours spent for both on-site and off-site activities, the implementation of guidelines in clinical practice and the utilization of the EMR including problems and barriers during its implementation.

#### Interviews and Audit

Apart from the above reports, the facilitators were scheduled to meet with the PCPs and the nurses at 6-month and 18-month follow-up visits in order to conduct semi-structured face-to-face interviews as well as focus group sessions at baseline and end of follow-up. The health professionals were expected to provide information on their overall satisfaction with the intervention, the experiences and potential barriers in the implementation of the study as well as their suggestions for improvements. At the end of the 6-month follow-up period, quality indicators audit were scheduled to be conducted in the intervention PCHCs. Additionally, semi-structured interviews of randomly selected patients from the intervention PCHCs were expected to take place at the end of the 18-month study period.

### Outcome Evaluation

The outcome evaluation of our intervention included the quality indicators for the selected illnesses along with validated instruments measuring patients' opinion. Quality indicators included specific and measurable elements of practice that can be used to assess the quality of care [40]. A set of quality indicators was developed by combining experts' opinion with current scientific evidence. Quality indicators for diabetes included fasting blood sugar, levels of HbA1c, blood pressure (BP), body mass index (BMI), lipid profile (TC, HDL-C, LDL-C, TG), microalbuminuria, fundal and feet examination, and prevalence of smoking. Hypertension quality indicators included: blood pressure measurement (SBP, DBP), BMI, lipid profile and prevalence of smoking.

In addition, we used the EUROPEP questionnaire, distributed to all patients from the intervention and control PCHC, to evaluate patients' opinion before and after the intervention [41]. The EUROPEP instrument is a reliable and internationally validated questionnaire that measures patients' satisfaction with respect to the care received and the interpersonal skills of primary care physicians. The Greek version was planned to be used in our study [42] after the appropriate cultural adaptation of the questionnaire as deemed appropriate for Cyprus.

### Statistical Analyses

Summary statistics were planned to be generated for baseline characteristics and clinical evaluations for each study arm. T-test and chi-square statistics were planned to be used to assess the homogeneity of study arms with respect to baseline characteristics. The primary outcome measure was the improvement in the quality indicators of patients with hypertension and diabetes assessed by three different statistical methods. The first was based on a comparison of patients found to be at target levels based on guideline recommendations, before and after the intervention, a comparison of quality improvement measurements using General Linear Model of Repeated Measures, and finally, the percentage of patients with more than 10% improvement over baseline in selected indicators at the end of follow up, as being consistent with previous work of Majumdar, et al [43].

Quantitative analyses of additional quality indicators included the following variables: monthly visits (total number of visits divided by the duration studied in months), and time to response measures (months until patient achieved target levels for specific indicators). Repeated measures were planned to be analyzed using mixed effects models. Correlations among measurements made on the same subject, were planned to be modeled using random effects and random regression coefficients, and through the specification of a covariance structure. All tests were planned to be two-sided and a level of statistical significance was set at 0.05. All study outcomes were planned to be analyzed on the basis of intention to treat.

Data obtained during the process evaluation would be qualitatively analyzed using audio tapes from face-to-face interviews. Focus groups and personal interview information would be transcribed and a framework approach analysis [44,45], was planned to be performed based on the five-step approach: familiarization, identifying a thematic framework, indexing, mapping and interpretation.

## Discussion

Many countries nowadays are facing financial constraints for health care expenditures. The appropriate design of cost-effective, country-specific QI interventions based on translational research [46] is one of the cornerstones of contemporary health care policy. In the current report we have presented the design of a multifaceted, country-specific and tailored to local practices pilot QI intervention in primary health care centers of Cyprus, grounded on a number of theoretical frameworks including the Unified Theory of Acceptance and Use of Technology, the Chronic Care Model, the Theory of Planned Behaviour and the Theory of Reasoned Action.

A broad array of key initiatives in improving the quality of primary care services such as national systems for inspection and monitoring of performance and pay-for-performance incentive programs have been extensively described in the recent medical literature in countries with a long tradition in quality improvement efforts [47]. Although such paradigms can be extremely useful, countries without past experience in QI interventions, as well as limited resources, may benefit significantly from examples originating from countries with similar experiences and comparable health care system parameters.

A number of limitations of our study design are worth noting. First, due to limited resources, the intervention was planned to be implemented in a small number of primary care centers with few physicians and nurses serving as evaluators, thus limiting our study's impact. However our findings could be indicative of the directionality of changes and possible improvements that were to be observed. Moreover, our study was not a randomized double-blind community-based controlled trial, since the PCHCs were not randomly selected. In addition, there was a broad diversity among the centers with respect to the population they served. Nevertheless, our before and after specific study design with concurrent controls provides sufficient validity. However, the power calculations of our pilot study are limited by the number of primary care centers participating in the intervention and control groups. Typically group randomized trials like ours, should include about 5 – 15 practices per group taking into account the intra-class correlation due to practice membership. Finally, although our multifaceted intervention may appears to be expensive, ample evidence suggests that the use of EMR and the improved care of patients with chronic conditions have the highest potential for cost savings. [48] Furthermore, examples in the medical literature suggest that the use of physician facilitators have favorable cost-benefit ratios when targeted at costly system issues. [49,50]

Our study is one of the first attempts to improve the quality of the primary care system of Cyprus based on contemporary methodological approaches and adoption of novel computerized technology. Our investigation was designed to explore issues of feasibility, acceptability from patients and health care professionals, and effectiveness of a pilot quality improvement intervention. In addition, our study was expected to evaluate the potential effect of theoretical frameworks on the implementation of multifaceted intervention programs in the primary care system of Cyprus and to what extend such theoretical frameworks can offer a safe base for the described objectives. Our study design could also provide the necessary theoretical model and applied methodology as well as the practical tools for future efforts towards universal EMR implementation and the management of chronic diseases based on standard guidelines in the primary care setting of Cyprus. However, the desired quality improvement will need to be implemented and evaluated beyond a pilot setting in order to provide firm evidence with respect to its effectiveness.

## Conclusion

The aim of our correspondence was mainly to illustrate the methodological approach in designing a multifaceted quality improvement intervention based on translational research in a country where applied research is limited. We believe that our design may lead to the implementation of a successful quality improvement intervention using relatively limited resources in an environment lacking previous QI attempts. The effectiveness evaluation of the intervention is expected to provide a strong basis for future efforts to craft a standardized approach for continuous quality improvement interventions in the primary care setting of Cyprus.

## Competing interests

The authors declare that they have no competing interests.

## Authors' contributions

CL conceived of the idea for the project, and supervised the project. CL, AP, GAS, TZ and HES developed the methodology. GAS supervised the project implementation and developed the data collection tools. All authors reviewed the methodological approach. GAS wrote the first draft of the manuscript, while all authors contributed to the final version of the manuscript. All authors read and approved the final manuscript.

## Pre-publication history

The pre-publication history for this paper can be accessed here:


